# Simultaneous Inhibition of MEK and Hh Signaling Reduces Pancreatic Cancer Metastasis

**DOI:** 10.3390/cancers10110403

**Published:** 2018-10-26

**Authors:** Dongsheng Gu, Hai Lin, Xiaoli Zhang, Qipeng Fan, Shaoxiong Chen, Safi Shahda, Yunlong Liu, Jie Sun, Jingwu Xie

**Affiliations:** 1 Wells Center for Pediatric Research, Department of Pediatrics, Indiana University School of Medicine, Indianapolis, IN 46202, USA; donggu@iu.edu (D.G.); zhang86@iu.edu (X.Z.); qifan@iu.edu (Q.F.); 2 Indiana University Simon Cancer Center, Indiana University School of Medicine, Indianapolis, IN 46202, USA; shahdas@iu.edu (S.S.); yunliu@iu.edu (Y.L.); 3 Department of Molecular and Medical Genetics, Indiana University School of Medicine, Indianapolis, IN 46202, USA; linhai@umail.iu.edu; 4 Department of Pathology and Laboratory Medicine, Indiana University School of Medicine, Indianapolis, IN 46202, USA; chen251@iupui.edu; 5 Division of Medical Oncology, Department of Medicine, Indiana University School of Medicine, Indianapolis, IN 46202, USA; 6 Departments of Medicine and Immunology, Mayo Clinic, Rochester, Minnesota, MN 55905, USA; Sun.Jie@mayo.edu

**Keywords:** hedgehog, metastatic niche, pancreatic cancer, Ihh, MEK

## Abstract

Pancreatic cancer, mostly pancreatic ductal adenocarcinoma (PDAC), is one of the most lethal cancer types, with an estimated 44,330 death in 2018 in the US alone. While targeted therapies and immune checkpoint inhibitors have significantly improved treatment options for patients with lung cancer and renal cell carcinomas, little progress has been made in pancreatic cancer, with a dismal 5-year survival rate currently at ~8%. Upon diagnosis, the majority of pancreatic cancer cases (~80%) are already metastatic. Thus, identifying ways to reduce pancreatic cancer metastasis is an unmet medical need. Furthermore, pancreatic cancer is notorious resistant to chemotherapy. While Kirsten RAt Sarcoma virus oncogene (K-RAS) mutation is the major driver for pancreatic cancer, specific inhibition of RAS signaling has been very challenging, and combination therapy is thought to be promising. In this study, we report that combination of hedgehog (Hh) and Mitogen-activated Protein/Extracellular Signal-regulated Kinase Kinase (MEK) signaling inhibitors reduces pancreatic cancer metastasis in mouse models. In mouse models of pancreatic cancer metastasis using human pancreatic cancer cells, we found that Hh target gene *Gli1* is up-regulated during pancreatic cancer metastasis. Specific inhibition of smoothened signaling significantly altered the gene expression profile of the tumor microenvironment but had no significant effects on cancer metastasis. By combining Hh signaling inhibitor BMS833923 with RAS downstream MEK signaling inhibitor AZD6244, we observed reduced number of metastatic nodules in several mouse models for pancreatic cancer metastasis. These two inhibitors also decreased cell proliferation significantly and reduced CD45^+^ cells (particularly Ly6G^+^CD11b^+^ cells). We demonstrated that depleting Ly6G^+^ CD11b^+^ cells is sufficient to reduce cancer cell proliferation and the number of metastatic nodules. *In vitro*, Ly6G^+^ CD11b^+^ cells can stimulate cancer cell proliferation, and this effect is sensitive to MEK and Hh inhibition. Our studies may help design novel therapeutic strategies to mitigate pancreatic cancer metastasis.

## 1. Introduction

Pancreatic cancer is the most deadly human cancer type [1]. Pancreatic cancer, now the third leading cause of cancer-related death, is predicted to become the second cause of cancer-related death by 2030 in the US (with lung cancer still the number one cause) [2]. Gemcitabine, approved to treat pancreatic cancer in 1997, continues to be the first line chemotherapy drug [3]. While most targeted and immune therapeutic strategies failed to benefit the patients, there are only two new strategies leading to slightly better patient survival, namely bi-weekly bolus plus infusional fluorouracil, leucovorin, irinotecan, and oxaliplatin regimen (Folfirinox) [4] and gemcitabine with Nab paclitaxel [5]. Thus, there is an urgent medical need to establish novel strategies to treat pancreatic cancer.

Previous studies show different roles of hedgehog signaling in pancreatic cancer. On the one hand, it is known that hedgehog (Hh) signaling is active during pancreatic cancer development and progression, and inhibition of Hh signaling allows better drug penetration in the primary tumor and reduces pancreatic cancer development [6,7,8]. Genetically, inactivation of Gli transcription factors slows down tumor development and progression of pancreatic cancer in the KrasLSL.G12D/+; p53R172H/+; PdxCretg/+ (KPC)-based mouse models. In KPC mouse models, Smoothened inhibitor IPI-926 was shown to reduce pancreatic cancer development [6]. On the other hand, genetic removal of *Shh* in pancreas or depletion of fibroblasts promotes pancreatic cancer development and progression in KPC-based mouse model [9,10]. These seemly contradicted results may be explained by the fact that both canonical and non-canonical Hh signaling exist during pancreatic cancer development and progression, and non-canonical Hh signaling is not affected by smoothened inhibitors. Failure of Smoothened inhibitors in clinical trials in patients with metastasis further confirms that inhibition of canonical Hh signaling alone is not sufficient to reduce pancreatic cancer progression, and indicates that paracrine Shh signaling has a very different role from Hh signaling in the cancer cells. Up to now, there are no reported combined therapeutics with smoothened inhibitor and another targeted therapeutic agent in cancer models, and this possibility may help re-initiate more clinical trials for novel cancer treatment.

K-RAS mutation is the most common genetic alteration in pancreatic ductal adenocarcinoma (PDAC) [11,12,13], and several mouse models of pancreatic cancer have been developed through inclusion of the most common K-RAS gene mutation K-RAS^G12D^ [14,15,16,17]. Currently, there are no specific therapeutic inhibitors for K-RAS although a number of inhibitors targeting RAS downstream effectors, such as MEK and phosphoinositide 3 kinase (PI3K), are available [11].

In this report, we tested the possibility that combination of smoothened inhibitor with an inhibitor targeting one of the K-RAS downstream effectors may be effective in reducing pancreatic cancer metastasis. In orthotopic mouse models using human pancreatic cancer cell lines, we found that Hh target gene *Gli1* is up-regulated during pancreatic cancer metastasis. Specific inhibition of Hh ligand-mediated signaling significantly altered gene expression profiles in the tumor microenvironment but had no significant effects on cancer metastasis. It is not known whether combining Smoothened inhibitors with inhibitors targeting K-RAS downstream effectors will be effective in suppression of pancreatic cancer metastasis. Both hedgehog signaling and K-RAS signaling are activated in pancreatic cancer. While Hh ligand-mediated signaling is mainly activated in tumor microenvironment, K-RAS is activated both in the cancer cells and in the tumor microenvironment. Targeting both pathways may produce a synergistic inhibition on pancreatic cancer metastasis. We have further delineated the mechanisms for the interactions between BMA833923 and AZD6144 using a variety of approaches. 

## 2. Results

### 2.1. Effects of Hh Signaling on Metastatic Niche Gene Expression

We first used an orthotopic mouse model for pancreatic cancer metastasis to monitor gene expression changes in the cancer cells and in the metastatic niche. Human MIA PaCa2 cells were used to form tumors in the pancreas of immune deficient NSG^tm^ mice, as initially established in Fidler’s laboratory and this model allows us to examine gene expression in the cancer cells (human gene transcripts) as well as in the metastatic niche (mouse gene transcripts). We also used mouse pancreatic cancer cells MMC18 [17] and Pan02 [18] in the metastatic models using immune competent C57/B6 mice for functional studies. In the metastasis mouse models, we ectopically expressed green fluorescent protein (GFP) and luciferase in cancer cells before spleen injection of the mice. As shown previously, these ectopically expressed proteins do not affect the metastatic characteristics and biology of pancreatic cancer cells, and we can monitor tumor growth by luciferase activity and the site of metastasis by the appearance of GFP expression [19]. We obtained the liver tissues with or without metastases for RNA extraction and gene expression analyses by real-time PCR and RNA sequencing. 

**Figure 1:**
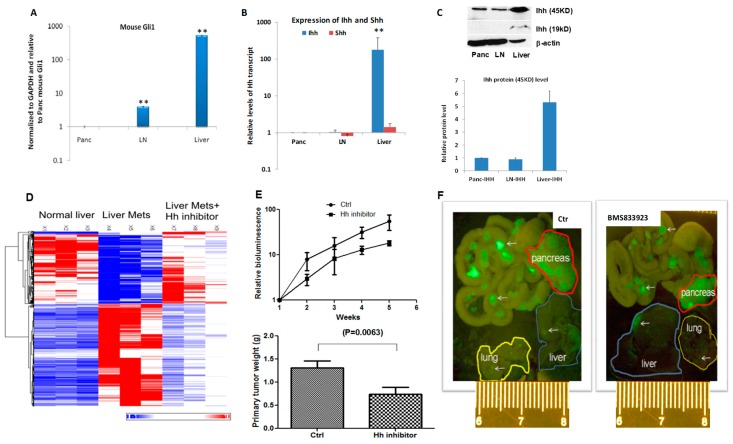
Activated hedgehog signaling in orthotopic mouse models of pancreatic cancer metastasis. (**A**) Expression of mouse *Gli1* gene, an indicator for hedgehog signaling activity, in tumor-bearing pancreatic tissues, metastatic lymph nodes and liver, was detected by Taqman-based real-time PCR analysis. ** *p* < 0.005 by Student *t*-test of two independent means (*n* = 5/group). (**B**) The transcript levels of *SHH* and *IHH* in different tissues were detected by real-time PCR (Taqman-based) ** *p* < 0.005 by Student *t*-test of two independent means (*n* = 5/group). (**C**) Western blotting analysis of IHH protein levels in tumors of pancreas, lymph nodes and liver. Metastatic nodules were dissected (based on GFP-expression) from liver tissues for protein analysis to reduce the Ihh protein level from the tumor microenvironment. We performed protein analysis from three independent mice with liver metastases and three normal liver tissues with similar results. (**D**) The effect of smoothened inhibitor BMS833923 on liver gene expression patterns in MIA PaCa2-based orthotopic mouse model was shown following RNA sequencing analyses. While metastatic liver tissues have a very different gene expression pattern from naïve liver tissues, addition of BMS833923 every the other day for 3 weeks altered the gene expression pattern to reassemble that of naïve liver tissues. (**E**) The effect of smoothened inhibitor BMS833923 on pancreatic cancer growth was revealed by weekly with bioluminescent intensity from the cancer cells as well as the tumor weight difference after the mice were sacrificed. Five mice per group were used in this experiment (with power 95% alpha error 0.05). (**F**) Whole body imaging detection of GFP expression pancreatic cancer cells in pancreas, lymph nodes, lung and liver in MIA Paca2-based orthotopic mouse model following administration of BMS833923 or with vehicle control. White arrows indicate the site of pancreatic cancer cells (GFP positive). Scale bar: centimeter. Similar results were also observed in AsPC1-based orthotopic mouse model (Figure S4 and S5).

We detected a high level of mouse *Gli1* transcript in the metastatic liver in comparison with that in the primary tumors or lymph node metastasis (Figure 1A, *p* < 0.005). As a hedgehog signaling target gene, high expression of *Gli1* indicates hedgehog signaling activation [20]. We also detected elevated human *GLI1* gene expression (Figure S1), indicating activated Hh signaling in the pancreatic cancer cells. 

To identify the molecular mechanism underlying hedgehog signaling activation, we examined expression of hedgehog ligands in the metastatic tumors as well as in the niche, and found elevated expression of *IHH* in cancer cells of metastatic liver tissues (Figure 1B,C, *p* < 0.005), indicating that inhibition of smoothened (e.g., BMS833923) may be effective in suppression of Hh signaling. Indeed, we found that the gene expression pattern in mice with liver metastasis was altered by treatment with smoothened inhibitor BMS833923 to resemble the gene expression pattern of the normal liver (Figure 1D), indicating that Hh signaling is important for gene expression regulation in the host.

To determine the significance of Hh signaling for pancreatic cancer metastasis, we treated the mice with pancreatic cancer metastasis with smoothened inhibitor BMS833923. After treatment, we examined the mice for pancreatic tumors, and the number/size of metastatic nodules. While we observed reduction of pancreatic tumor size (Figure 1E, *p* < 0.05), metastatic nodules were not significantly affected (see Figure 1F for representative images). This is not surprising, and is consistent with the failed clinical trial with smoothened inhibitors vismodegib [21,22] and saridegib [23] in patients with metastatic pancreatic cancer. 

Smoothened inhibitor BMS833923 mainly blocks Hh ligand-mediated signaling in tumor microenvironment [19]. In contrast, K-RAS signaling is activated both in the pancreatic cancer cell and in the tumor microenvironment. While the single agent BMS833923 was not effective in suppression of pancreatic cancer metastasis, it is still possible that combining hedgehog signaling inhibition with reduction of K-RAS signaling, the major driver for pancreatic cancer development, may be effective. We have evidence to show that inhibition of K-RAS downstream effector MEK signaling by AZD6244, but not the PI3K signaling by BEZ235, reduced Hh target gene *GLI1* expression in pancreatic cancer cells (Figure S2). We thus predict that combination of MEK inhibitor AZD6244 with Smoothened inhibitor BMS833923 may be effective in suppressing pancreatic cancer metastasis.

### 2.2. Suppression of MEK/ERK and Hh Signaling Reduces Pancreatic Cancer Metastasis

While there are several mouse models for pancreatic cancer metastasis, therapeutic studies require robust models to obtain data with sufficient power. We decided to use two models to test the effects of combined MEK/Hh signaling inhibitors. First, orthotopic mouse models using human pancreatic cancer cells in immune deficient mice are feasible for these studies, enabling us to determine the effects of drugs on human cancer cells. Second, one commonly used model for liver metastasis of pancreatic cancer is to inject cells into spleen, and to examine the number of metastatic nodules in the liver a few weeks later [24,25,26]. For all mouse cell lines tested in the liver metastasis model, we observed liver metastasis in all mice and the time of onset does not vary very much from mouse to mouse (e.g., ~20 days for MMC18 cell-based model). In contrast, several GEM models are not feasible for drug treatment studies of metastasis. First, the metastasis onset and mouse survival time vary significantly from mouse to mouse in several GEM models with spontaneous liver and lung metastasis, including the KPC model, Pdx1-cre/KRAS^G12D^/p16^F/F^ mice [14,17,27]. For example, KPC mice have metastasis onset from 10 to 40 weeks (our unpublished observation and Hingorani et al. [14]), making it hard to compare the number of metastatic nodules in different groups. Similarly, Pdx1-cre/KRAS^G12D^/p16^F/F^ [17] mice have metastatic onset from 8 to 20 weeks (our observation and Qiu et al. [17]), and the power for distinguishing the difference between the two groups requires more than 100 mice in each group, which is not feasible. Second, pancreatic injection of mouse pancreatic cancer cell lines (KPC2, MMC18 and Pan02) leads to quick development of pancreatic tumors but rarely have liver and lung metastases. 

**Figure 2:**
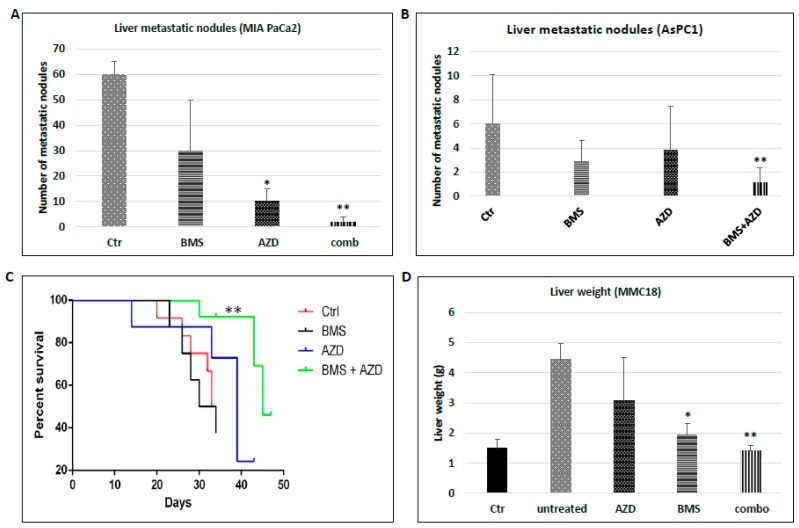
Simultaneous inhibition of MEK and Hh signaling in mouse models. (**A**) The number of metastatic nodules in the liver in MIA PaCa2-based orthotopic model in immune deficient NSG mice from four different treatments: (1) treated with smoothened inhibitor BMS833023; (2) treated with MEK inhibitor AZD6244; (3) the combination of AZD6244 and BMS833923; and (4) vehicle control (5) mice/group); (**B**) Effect of different treatments on the number of metastatic nodules in AsPC1-based mouse model (5 mice/group); (**C**) Kaplan-Meier survival curve for mice with different treatments in AsPC1-based mouse model, ** *p* < 0.05 was derived from Log-rank (Mantel-Cox) test; (**D**) Effect of different treatments on liver tissue weight, a measurement for liver metastasis, in MMC18-based model in immune-competent C57/B6 mice (5 mice/group). * *p* < 0.05 and ** *p* < 0.005 indicate significant or synergistic effect by BLISS independence analysis (see Statistical analyses for details).

We first tested the effect of the combined treatment with MEK and Hh inhibitors in orthotopic mouse models using human cancer cells. MIA PaCa2 cells were used for orthotopic models in immune deficient NSG mice. Five days after pancreatic injection when microscopic metastasis is observed in the liver (as indicated by GFP positive cells in intravital microscope images in Figure S3), we treated mice with Hh inhibitor BMS833923, MEK inhibitor AZD6244, the combined treatment, or with PBS control. As shown in Figure 2A, we found that the combined treatment led to a significant decrease in the number of metastatic nodules in the liver (see Figure S4 for GFP positive nodules). We calculated the number of mice with liver metastasis in different groups, and found that the mice treated with both AZD6244 and BMS833923 had no or very few visible metastatic nodules whereas mice in other groups all had over 10 metastatic nodules in the liver. In contrast to metastatic nodules, we did not observe any synergy between AZD6244 and BMS833923 in reduction of tumor weight in this orthotopic model (Figure S5). Similar results were also obtained using AsPC1 cells (Figure 2B). In addition, we found that the lifespan in the double treatment group was significantly extended in the AsPC1-based mouse model (Figure 2C). For MIA PaCa2 based model, mice can survive longer than 2 months without any observed changes in survival for reasons we still do not understand. In summary of this experiment, we observed a synergistic effect (as expressed in statistical term as a more than additive effect) between BMS833923 and AZD6244 in two orthotopic mouse models.

Next, we tested the effects of drug treatments using mouse cell line MMC18 in immune competent mice [17,19]. We treated the mice 2 days after spleen injection when microscopic metastases are detectable. This way, we can determine whether inhibition of MEK and Hh signaling is sufficient to reduce an important step in metastasis, post-extravasation tumor growth (colonization). Due to appearance of numerous metastatic nodules in the liver in this model (*n* > 100), we used liver weight as a way to measure the severity of liver metastasis instead, which has been previously used by many other researchers [18,28,29]. We observed that the liver weight was reduced by the combined treatment with MEK and Hh signaling inhibitors, and the effect was more than additive, suggestive of a synergistic effect between BMS833923 and AZD6244 according to BLISS independent analysis (see Methods for this analysis) in comparison with single treatments (Figure 2D). The compounds for MEK and Hh signaling inhibition are very specific. For example, BMS833923 reduced the expression of *Gli1* over 90% (Figure S6A). Similarly, AZD6244 reduced the ERK phosphorylation significantly (Figure S6C,D). These results indicate that inhibiting hedgehog and MEK signaling is sufficient to reduce metastatic colonization of the cancer cells in the liver.

Taken together, our data indicate that simultaneous suppression of MEK and Hh signaling has a more than additive effect on reduction of metastasis, particularly the post-extravasation tumor growth of pancreatic cancer, whereas single agents have limited effects. 

### 2.3. Effects of MEK Inhibitor AZD6244 and Smoothened Inhibitor BMS833923 on Cell Proliferation

**Figure 3:**
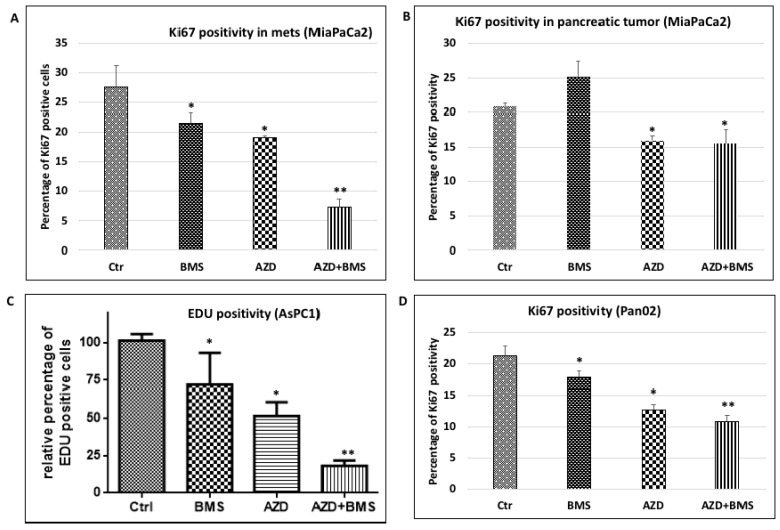
Effects of simultaneous inhibition of MEK and Hh signaling on cell proliferation. (**A**) Effect of MEK/Hh signaling inhibition on Ki-67 positivity in metastatic liver tissues of MIA PaCa2-based model in immune-deficient NSG mice; (**B**) The rate of Ki-67 positivity in pancreatic cancer of MIA PaCa2-based model after different treatments; (**C**) Percentage of EdU labeling after different treatments in AsPC1-based model in immune deficient NSG mice; (**D**) Effect of different treatments on Ki-67 positivity in Pan02-based model in immune competent C57/B6 mice. The percentage of Ki67 positivity was derived from the average positivity of at least three independent mice, and the average was obtained from five fields of immunofluorescently stained slides. * indicates *p* < 0.05 and ** indicates a synergistic (more than additive) effect via BLISS independence analysis.

The post-extravasation tumor growth of pancreatic cancer may be caused by increased cell proliferation or decreased apoptosis (or cell death). Because we did not observe any significant changes in the level of cleaved caspase 3 (an indicator for apoptosis) by combined treatment with AZD6244 and BMS833923, we turned our focus on regulation of cell proliferation by these two inhibitors. We detected Ki-67 staining or EdU labeling to determine changes in cell proliferation. Because both MEK signaling and the Hh pathway are known to regulate cell proliferation [30,31,32,33], we predict that both AZD6244 and BMS833923 should reduce the rate of Ki-67 positivity. As shown in Figure 3A (MIA PaCa2- based model), Ki-67 staining positivity in the untreated metastatic liver tumor was 27.57%. AZD6244 treatment reduced Ki-67 positivity to 19.13% whereas BMS833923 treatment resulted in 21.51% Ki-67 positivity. Furthermore, combined treatment with AZD6244 and BMA833923 reduced Ki-67 positivity to only 7.25%, which is significantly more effective than single treatments. According to BLISS independence analysis, the effect from these two drugs is synergistic (more than additive effect in statistical term, as indicated by ** in Figure 3). In contrast to the metastatic tissues, we did not observe a synergistic effect between AZD6244 and BMS833923 in cell proliferation of the primary pancreatic tumors (Figure 3B). We also observed a synergistic effect between AZD6244 and BMS833923 on reduction of Ki-67 positivity in AsPC1 and MMC18-based mouse models (Figure 3C,D).

To determine how MEK inhibitor AZD6244 can have a synergy with Smoothened inhibitor BMS833923, we have performed gene expression analysis in metastatic tumor microenvironment. As shown in Figure S7, we found that both MEK inhibitor AZD6244 and Smoothened inhibitor BMS833923 affected gene expression in the metastatic niche. We further discovered that the top three pathways affected were also shared by these two inhibitors, including leukocyte extravasation signaling, agranulocyte adhesion and diapedesis, and hepatic fibrosis. These analyses indicate that both BMS833923 and AZD6244 affect cell functions in the metastatic niche.

### 2.4. Effects of MEK Inhibitor AZD6244 and Smoothened Inhibitor BMS833923 on Cell Population at the Metastatic Niche

**Figure 4:**
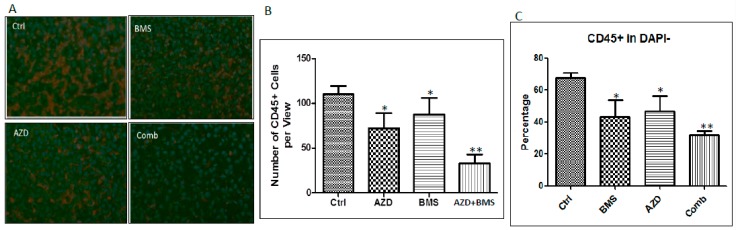
Regulation of CD45^+^ cell number by both MEK and Hh signaling pathways. (**A**) Representative immunofluorescent staining images of CD45^+^ cells in liver tissues after drug treatments. (**B**) Summary of **A** from 10 images in each group (from at least 3 mice) with the number of CD45^+^ cells per field under microscope (200×). (**C**) Summary of flow cytometry analysis of CD45+ cells in different treatment groups (from three mice/group). * indicates *p* < 0.05 and ** indicates a more than additive effect via BLISS independence analysis.

We prepared single cells from metastatic tissues (mainly liver), and performed flow cytometry analyses. We observed a significant increase in CD45^+^ cells, particularly Ly6G^+^ cells (Figure 4A and Figure S8). We assessed whether the percentage of CD45 cells in the liver is regulated by MEK and Hh signaling inhibitors. The number of CD45 cells in the metastatic liver was measured after treatment with these two inhibitors by immunofluorescent staining (Figure 4A,B) and flow cytometry (Figure 4C). As shown in Figure 4A,B, we found that the number of CD45^+^ cells was reduced by AZD6244 as well as by BMS833923, and the combined treatment had a more significant reduction based on BLISS independence analysis, as shown in Figure 4B [34]. We also confirmed this effect by flow cytometry analysis (Figure 4C). 

**Figure 5:**
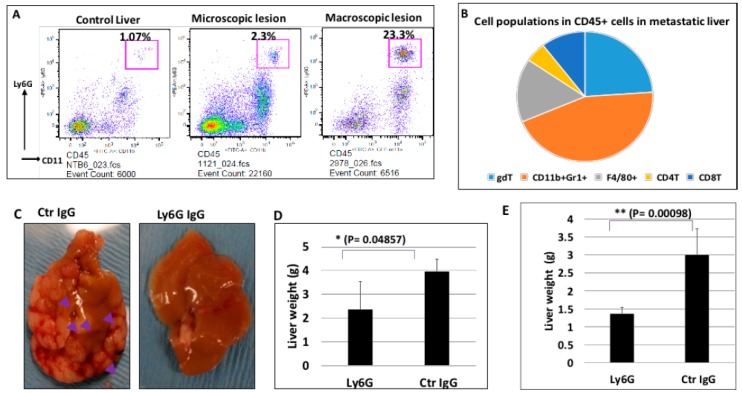
Effects of MEK/Hh signaling inhibition on Ly6G^+^CD11b^+^ cells in the metastatic liver tissues (MMC18-based model in immune competent C57/B6 mice). (**A**) Representative images of flow cytometry of CD45^+^CD11b^+^Gr1^+^ cells in different liver tissues; (**B**) Cell population composition in metastatic liver tissues, and the number was the average from five mice/group; (**C**) Representative images of liver tissues with or without treatment of Ly6G neutralizing antibodies. While IgG treated mice had many metastatic nodules (indicated by purple arrowheads), Ly6G neutralizing antibody treatment led to no visible or only a few metastatic nodules; (**D**) Ly6G neutralizing antibody treatment led to reduced liver weight in MMC18- based mouse model (five mice/group), suggesting a reduced liver metastasis; (**E**) Similar results were obtained using Pan02 cells (five mice/group). Ly6G neutralizing antibody treatment led to reduced liver weight. * *p* values were calculated using Student *t* test of two independent means.

Furthermore, we analyzed different populations in the CD45^+^ cells, and found that CD11b^+^Gr1^+^ cells (with majority of them Ly6G^+^CD11b^+^ cells) are the most common cell type in the metastatic niche by flow cytometry analysis (Figure 5A,B) [35,36,37], suggesting that these cells may be critical for metastatic colonization of pancreatic cancer cells. CD11b^+^Ly6G^+^ cells may be neutrophils or myeloid-derived suppressor cells (MDSC). Because both cell types have similar surface markers. It is also possible that this is a mixed cell population of neutrophils and MDSC.

### 2.5. The Significance of Ly6G^+^CD11b^+^ Cells for Liver Metastasis of Pancreatic Cancer

We have evidence to show that Ly6G^+^CD11b^+^ cells are the cell population affected most by combined treatment with BMS833923 and AZD6244 (Figure S9). To determine the significance of Ly6G^+^CD11b^+^ cells for liver metastasis of pancreatic cancer, we used specific neutralizing antibodies to deplete Ly6G^+^ cells in two mouse models: MMC18-based model through injection of matrix gel-embedded cancer cells into spleen; Pan02 cells as another model [29,38,39]. The host mouse is immune competent C57B/6. After cell injection, we treated mice with Ly6G neutralizing antibody (clone 1A8) or the control IgG protein (see Methods for details). The effect of 1A8 IgG was assessed by the appearance of the Ly6G^+^CD11b^+^ cell population after administration of antibodies. Reduction of the CD11b^+^Gr1^+^ cell population by 70% with 1A8 IgG will indicate successful cell depletion (Figure S10). We found that 3 out 4 mice treated with 1A8 IgG reduced the Ly6G^+^CD11b^+^ cell population by 70% in the MMC18 mouse model. In the Pan02 mouse model, all mice treated with 1A8 IgG had reduced Ly6G^+^CD11b^+^ population by 70%. Four weeks after injection, we measured the liver weight to show the severity of liver metastasis [18,28,29]. As shown in Figure 5 (Figure 5C,D), Ly6G neutralizing antibody-treated mice had significantly reduced liver weights in comparison with the control group. While depletion of immune cells does contribute to the reduced liver weight (by ~10%), reduction of liver weight by ~50% indicates a major effect of Ly6G^+^CD11b^+^ cells on metastasis. We obtained similar results from Pan02-based model (Figure 5E). These results indicate that Ly6G^+^CD11b^+^ cells are critical for liver metastasis of pancreatic cancer. 

### 2.6. Stimulation of Cancer Cell Proliferation by Ly6G^+^CD11b^+^ Cells

**Figure 6:**
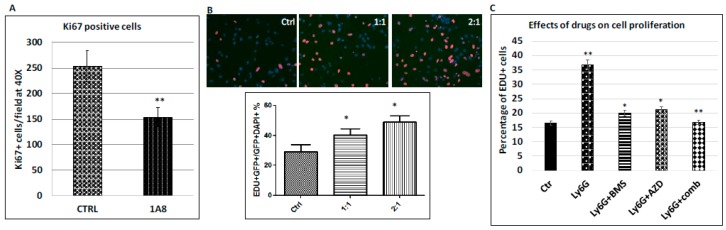
Regulation of cancer cell proliferation by inhibition of MEK and Hh signaling in mouse models and in cultured cells. (**A**) Expression of Ki-67 was used as the readout of cell proliferation, and percentage of Ki-67 positive cells in the metastatic cancer cells was calculated under microscope from MMC18-based model in immune competent C57/B6 mice (five mice/group), * *p* value < 0.05. These results are similar to those from MIA PaCa2-based model in immune deficient NSG mice (see Figure 3A); (**B**) EdU incorporation of cancer cells in the presence or absence of Ly6G^+^CD11b^+^ cells in vitro. EdU incorporation of cancer cells was used to measure cell proliferation rate. With an increase in Ly6G^+^CD11b^+^ cells (ratio 0:1; 1:1; 1:2 with increased number of Ly6G^+^CD11b^+^ cells), EdU incorporation in the cancer cells was visualized (representative images shown above; summary from 3 independent experiments shown below), scale: 400×; (**C**) Effects of different compounds in reducing cell proliferation of cancer cells were assessed in vitro. Ly6G^+^CD11b^+^ cells were first treated with different compounds for 2 h before adding to the top chamber. The data were from three independent experiments. * indicates significant change (*p* < 0.05). While Ly6G^+^CD11b^+^ cells significantly stimulated cell proliferation of cancer cells (* indicates *p* < 0.005), treatment with MEK inhibitor AZD6244 (or Hh signaling inhibitor BMS833923) for 2 hours before being added onto the top chamber significantly reduced cancer cell proliferation (*p* < 0.05). Combination of AZD6244 with BMS833923 had a more than additive effect on cell proliferation (** indicates synergistic effect via BLISS independent analysis).

The major feature of cancer cell colonization is an increase in proliferation of metastatic cancer cells. Using Ki-67 positivity as a marker for cell proliferation, we found that MEK and Hh signaling inhibition simultaneously reduces proliferation of the metastatic cancer cells in the mouse model (Figure 3). Similarly, we investigated whether Ly6G neutralizing antibodies affect proliferation of metastatic cancer cells, and we measured cell proliferation using Ki-67 staining in liver tissues treated with either Ly6G neutralizing antibodies or the control IgG in the mouse models. As indicated in Figure 6A, we found that Ki-67 positive cancer cells (K19^+^) was significantly reduced (*p* < 0.005). These data indicate that the metastatic niche cells, such as Ly6G^+^ cells, can promote cancer cell proliferation.

To determine whether Ly6G^+^ cells promotes cancer cell proliferation through direct cell-cell interaction or indirectly through secretion of growth factors, we used inserts to separate cancer cells from Ly6G^+^CD11b^+^ cells. The pore size of the inserts was small enough (0.45 M) to prevent Ly6G^+^CD11b^+^ cells from slipping through the pore. First, we tested EdU incorporation of cancer cells in the presence of equal amount of Ly6G^+^CD11b^+^ cells, or at 2:1 and 4:1 ratio (Ly6G^+^CD11b^+^ cells vs. cancer cells). As shown in Figure 6B, we found that a significant increase of the EdU incorporation rate by addition of increased amount of Ly6G^+^CD11b^+^ cells, indicating that Ly6G^+^CD11b^+^ cells indeed can stimulate cancer cell proliferation, and this effect was cell-contact independent.

In the next experiment, we tested whether combination of AZD6244 and BMS833923 can synergistically suppress cancer cell proliferation. Similar to the animal studies, we had four groups of samples: BMS833923-treated group, AZD6244-treated group, the combined treatment group and the untreated control. We found that while both drugs reduced the 5-ethynyl-2′-deoxyuridine (EdU) incorporation rate of the cancer cells, the combined treatment group had a more than additive effect on cancer cell proliferation (BLISS independence analysis), making the EdU incorporation rate nearly back to the basal level (Figure 6C). These results are very similar to the animal studies, indicating that Ly6G^+^CD11b^+^ cells are the major driving force for elevated cell proliferation of metastatic cancer cells, which is essential for metastatic colonization of pancreatic cancer cells in the liver. These results indicate that Ly6G^+^CD11b^+^ cells are critical for cell proliferation of the metastatic cancer cells, and this effect requires MEK and Hh signaling. 

In summary, we discovered that combined inhibition of Hh signaling and MEK signaling reduces pancreatic cancer metastasis in mouse models. Simultaneous inhibition of these two signaling pathways suppressed metastatic colonization of pancreatic cancer cells. We identified Ly6G^+^CD11b^+^ cells as the major cell population targeted by both pathways, and depletion of this population significantly reduced colonization of pancreatic cancer cells in the liver. We further demonstrated that Ly6G^+^CD11b^+^ cells are able to stimulate proliferation of cancer cells in vitro, and suppression of MEK and Hh signaling synergistically reduced this effect. These results suggest that combination of a smoothened inhibitor with a MEK inhibitor may be effective in clinical management of metastatic pancreatic cancer.

## 3. Discussion 

Progression and metastasis of pancreatic cancer is accompanied with numerous changes in the cancer cells as well as in the metastatic niche, and mouse models have provided the means to study the significance of these changes [9,10,40,41,42,43,44,45,46]. Alteration in the metastatic niche can also offer an opportunity in novel therapeutics [47]. Our data have demonstrated that combining smoothened inhibitor BMS833923 with MEK inhibitor AZD6244 is more effective in suppression of pancreatic cancer metastasis, as indicated by reduced the number of metastatic nodules (Figure 2), a decrease in cell proliferation (Figure 3) and reduced number of Ly6G^+^CD11b^+^ neutrophils (Figure S8). It appears that MEK signaling regulates both cancer cells and the metastatic niche. We have further shown that depletion of Ly6G^+^ cells is also effective in reducing the number of metastatic nodules (Figure 5). These data provide a rationale to combine smoothened inhibitors with MEK inhibitors for clinical trials in pancreatic cancer patients. 

Although the molecular mechanism underlying Hh signaling activation in the metastatic niche is currently not well characterized, our data suggest that elevated *IHH* expression in the metastatic cancer cells may be responsible (Figure 1). We have shown both in the mouse model and in human specimens that *IHH* is highly expressed in the metastatic cancer cells (Figure 1). If IHH is the major driver for Hh signaling activation, neutralizing antibodies to IHH should be effective in suppressing Hh signaling in the niche as well as liver metastasis of pancreatic cancer. 

Ly6G^+^CD11b^+^ cells are major myeloid-derived immature cells, often with T-cell suppressive effect. They could be neutrophils. This is the very cell population with the most significant change in the metastatic niche in the mouse models, both in immune deficient mice and in immune competent mice (Figure 5 and Figure S7). In our study, we found that this cell population has a significant proliferation-promoting effect on cancer cells both in vitro co-culture experiment and in mice (Figure 6). In immune competent mice, we also observed that depletion of Ly6G^+^/CD11b^+^ cells increases CD8^+^ T cell population (Figure S8), consistent with a T-cell suppressing role. Thus, our studies indicate that Ly6G^+^/CD11b^+^ cells are a cell population with multiple cellular functions. Due to their significant changes in metastases, strategies to suppress their function in the human pancreatic cancer patients will have a significant clinical implication on cancer treatment. 

We have shown that combination of smoothened inhibitor BMS833923 and MEK inhibitor AZD6244 synergistically reduced liver colonization of pancreatic cancer cells. Colonization, described as a process of the post-extravasation tumor growth, is a limiting step for metastasis. At present, the exact factors driving tumor cell proliferation during colonization of pancreatic cancer remain to be identified. Our evidence indicates that PDGF signaling is important for proliferation of metastatic pancreatic cancer cells. PDGF signaling is a known signaling pathway involved in cancer cell proliferation [48,49,50,51,52]. For example, we showed that purified Ly6G^+^/CD11b^+^ cells induce proliferation of cancer cells (as indicated by an increase in EDU labeling) (Figure 5). When PDGF-A neutralizing antibodies were used, the increase of cell proliferation in the cancer cells was significantly inhibited. At the same time, addition of PDGF-A neutralizing antibodies alone had no effects on cancer cells, suggesting that these neutralizing antibodies affect cancer cells through Ly6G^+^/CD11b^+^ cells. We also noticed that PDGF-A neutralizing antibodies were not as effective as the combination of MEK/HH inhibitors, indicating that PDGF-A is not the only factor stimulating cancer cell proliferation. 

## 4. Materials and Methods

### 4.1. Chemicals

Hh signaling inhibitors BMS833923 was provided by Bristol–Myers Squibb (New York, NY, USA). BMS833923 is a potent synthetic small molecule (EC50 = 50 nmol/L) with specific inhibition on smoothened signaling (18). BMS833923 was originally patented by Exelixis (Alameda, CA, USA) and is now licensed to Bristol–Myers Squibb. AZD6244 was purchased from the Selleckchem Chemicals LLC (Houston, TX, USA).

### 4.2. Cell Lines

AsPC1 & MIA PaCa2 were purchased from American Type Culture Collection (ATCC, Manassas, VA, USA), authenticated by STR profiling, and cultured as instructed by the vendor. Pan02 was purchased from ATCC. All culture materials were purchased from Gibco/Life Technology Inc., (Gibco, Grand Island, NY, USA). MMC18 cells, provided by Dr. Gloria Su, were generated from metastatic tumors of mouse pancreatic cancer model [17], and cultured in Dulbecco’s Modified Eagle’s Medium (DMEM) with 10% fetal bovine serum (FBS). AsPC1 was cultured in RPMI1640 Medium supplemented with 10% heat-inactivated fetal bovine serum, 100 U/mL penicillin and 100 mg/mL streptomycin. Other cell lines were cultured in Dulbecco’s Modified Eagle’s Medium (DMEM) supplemented with 10% heat-inactivated fetal bovine serum (Gibco, Grand Island, NY, USA), 100 U/mL penicillin and 100 mg/mL streptomycin. The cultures maintained in a humidified incubator at 37 °C and 5% CO_2_.

### 4.3. Animals 

NOD/SCID/IL2R^null^ (NSG) female mice were provided by the In Vivo Therapeutics (IVT) Core in the Simon Cancer Center at Indiana University School of Medicine. C57Bl/6 female mice were purchased from Jackson laboratory. Use of animal was approved by the IACUC committee in Indiana University School of Medicine (ethical code 11370; approval date—15 February 2018).

### 4.4. Orthotopic Mouse Model of Pancreatic Cancer Metastasis 

AsPC-1 and MIA PaCa2 cells with stable expression of GFP and luciferase were harvested in single cell suspension at a concentration of 4 × 10^6^ cells/ ml as described in a previous publication [19]. A total of 2 × 10^5^ cells (in 50 l of growth medium) were injected into pancreas of NSG mice using a 27-gauge needle according to a protocol developed in Fidler’s laboratory [25]. C57/B6 mice were anesthetized and spleens were exposed. Pan02 and MMC18 cells in 50 μL of growth factor-reduced Matrix gel (BD Bioscience, San Jose, CA, USA) was injected into spleen to establish a liver metastasis model as reported previously [18]. Drug treatment started five days after tumor inoculation. BMS-833923 was dissolved in acidified water (9.99 mL sterile water plus 10 uL 1N HCl) at concentration 7.5 mg/mL. AZD6244 was suspended in sterile PBS by sonication at 5 mg/mL. For drug treatment, mice were treated with BMS-833923 (oral gavage at 15 mg/Kg body weight daily), AZD6244 (oral gavage 10 mg/kg daily) or corresponding control vehicles in each group. More precise tumor location and tumor size were confirmed. After mouse was dissected, tumor weight was measured and metastatic tumors were located. Tumor lesions in pancreas, liver, lung and lymph nodes were harvested and divided into several portions. One portion was snap-frozen in liquid nitrogen for mRNA extraction; some were fixed in 10% buffered formalin and embedded in paraffin for H&E staining and immunohistochemistry; others fixed in zinc fixative buffer and embedded in paraffin for immunofluorescence staining of CD45.

### 4.5. Depletion of Ly6G^+^ Cells 

Rat anti-Ly6G (1A8) antibody and control Rat IgG were generously given by Dr. Jie Sun. Mice received 50 μg either anti-Ly6G antibody or control IgG in PBS in a volume of 200 μL injected intraperitoneally beginning on the same day of cancer cells inoculation and continuing twice a week throughout the study.

### 4.6. Quantification of Metastatic Burden 

The number of visible lymph node, liver and lung metastases was determined with aid of whole body imaging, and lung metastases were also confirmed microscopically. Metastatic nodules on liver surface were counted macroscopically in MIA PaCa2 injected mice as previously reported [53,54]. It was counted as 100 if the number of metastatic nodules is above 100. In Pan02 and MMC18 injected mice, metastatic foci are difficult to be counted due to tumor immergence, so liver weight was used as an indicator to evaluate metastatic burden [18].

### 4.7. Single Cell Isolation 

Using a 21-blade scalpel, mince the tumor-containing liver tissues into the smallest possible fragments (less than 1 mm^3^) and place the tumor into 50 mL conical tube containing 10 mL collagenase IV (Worthington, Lakewood, NJ, USA) solution at a concentration of 1 mg/mL. Place the tube(s) into a 37 °C water bath with shaking for 1–1.5 h, agitating with a 10-mL pipette every 20 min to augment digestion. Then the digested tumor solution was strained through a sterile 70-μm cell strainer (BD Falcon, San Jose, CA, USA), and the resulting cell solution was collected and washed 3 times followed by cell number counting and antibody labeling for flow cytometry. 

### 4.8. Immunohistochemistry (IHC) and Immunofluorescence (IF) 

Fresh tissue was harvested and fixed with 10% buffered-formalin or zinc-based fixative [24], which was used for surface marker staining of infiltrated blood cells in tissue, such as CD45. Five-micron paraffin-embedded sections were labeled with primary antibodies against phosphor-p44/p42 MAPK (Erk1/2) (Thr 202/Tyr 204) (Cell Signaling Technology Cat# 4370, 1:200, Danvers, MA, USA), Ki-67 (ab15580, 1:500, AbCam, Cambridge, MA, USA) and CD45 (14-0451, 1:100, eBioscience, San Diego, CA, USA). 

### 4.9. Flow Cytometry

Single cells from primary and metastatic tumors were incubated with fluorescence conjugated antibody against mouse CD45, Ly6G, CD11b, Ly6C, F4/80 (Biolegend, San Diego, CA, USA) for 30 min in 4 °C. After washed, cells were stained with 1ug/ml DAPI for discrimination of dead cells. Flow cytometry was performed on a LSR407 device (Beckton Dickinson, Franklin Lakes, NJ, USA) and data analyzed using flowjo software. For cell sorting, stained cells were sorted on a BD FACSAria (BD, San Jose, CA, USA) according to the fluorescent colors used.

### 4.10. Cell Proliferation Assay 

Cell proliferation assay was performed in a double chamber co-culture system. MIA PaCa2 or MMC18 cells were seeded in 24-well plate with cover slip at the density of 20,000 cells/well. Next day, cells were starved DMEM medium containing 0.5% FBS for 6 hours before co-cultured with sorted neutrophils. CD45^+^CD11b^+^Ly6G^+^ cells sorted from metastatic tumor bearing liver were seeded in the upper chamber (0.4 um transwell) at different ration to cancer cells in 0.5% FBS DMEM for 24 h. 2 h before collection of the cancer cells, ethynyl-2-deoxyuridine (EdU) was added in lower chamber at 5 uM final concentration. Edu incorporated into DNA was detected according to the protocol of the manufacturer (Click-iT^®^ EdU Alexa Fluor^®^ 594 Imaging Kit; Invitrogen, Carlsbad, CA, USA). To evaluate cell proliferation in vivo, mice were injected with EdU intraperitoneally at 40 mg/kg, 4 h before being sacrificed. We visualized the incorporation of EdU on tissue slides to identify cells undergoing DNA replication as previously reported [55].

### 4.11. RNA Extraction, RT-PCR and Real-Time PCR

Total RNAs of cells were extracted using Tri-RNA reagent from Sigma according to the manufacturer’s instruction and 1 μg of total RNA was reverse transcribed into cDNAs using the first-strand synthesis kit (Roche, Tucson, AZ, USA). Real-time quantitative PCR analyses were performed according to a previously published procedure. Triplicate C_T_ values were analyzed in Microsoft Excel using the comparative *C*_t_(ΔΔ*C*_t_) method as described by the manufacturer (Applied Biosystems, Foster City, CA, USA). The amount of target (2^−^^ΔΔ*C*t^) was obtained by normalization to an endogenous reference (*Gapdh* for mice and *GAPDH* for humans) and relative to a calibrator. All taqman primers and probes were purchased from Applied Biosystems Inc. and the catalog numbers are shown below (Table 1):

### 4.12. RNA-seq, Sequence Alignment, Differential Expression Analyses

For transcriptomic analysis, total RNAs were extracted using TRIzol Reagent (Invitrogen), and the RNA quality in each specimen assured by the Agilent bioanalyzer profiles before moving on to cDNA synthesis and library construction. Sequencing was conducted using SOLiD 5500 with 75 bp in one direction. Analyzing process was conducted as the normal bioinformatics analyzing method. The RNA abundance was evaluated by Reads per kilobases per million reads (RPKM). All sequenced libraries were mapped to the mouse genome and human genome separately (UCSC mm9, hg19) using LifeScope 2.5 with the default parameters. The reads distribution across the genome was assessed using bamutils (from ngsutils) [56]. Sequencing reads which can be uniquely mapped to either genome with mapping quality >8 were kept for further analysis. Reads that mapped to each genome were assigned to mm9 refGene and hg19 refGene genes respectively. Genes with read count per million (CPM) < 1 in more than half of the samples were removed. The data was normalized using TMM (trimmed mean of M values) method. Differential expression analysis was performed using edgeR with generalized linear model [57,58]. False discovery rate (FDR) was computed from *p*-values using the Benjamini-Hochberg procedure. The ingenuity pathway analysis (IPA) was performed on genes that significantly (adjusted *p*-value < 0.05) differentially expressed between two groups.

### 4.13. Western Blot Analysis 

Aspc1 cells bearing tumor tissue from pancreas, lymph node and liver was lysed in cell lysis buffer (50 mM Hepes, pH 7.4, 2 mM EDTA, 100 mM NaCl, 1% Glycerol, 1% Triton X-100) containing protease and phosphatase inhibitors (Roche). Added 200 µL of ice-cold lysis buffer per 10 mg tissue and homogenized with an electric homogenizer on ice. After centrifuge, supernatant was used for analysis. After separation by 8% or 10% SDS-polyacrylamide gel electrophoresis and protein transfer onto PVDF membrane. Expression of proteins was detected using the following antibodies: IHH (AbCam, ab39634, 1:500, Cambridge, MA, USA); phosphor-p44/p42 MAPK (Erk1/2) (Thr 202/Tyr 204) (Cell Signaling Cat# 9101, 1:1000, Danvers, MA, USA). Protein loading was assessed by the presence of total p44/p42 MAPK (Erk1/2) (Cat# 9102, 1:1000, Cell Signaling Technology Inc., Danvers, MA, USA) and beta-actin (Cat# A5441, 1:10,000, Sigma, St. Luis, MO, USA). 

### 4.14. Statistical Methods 

Data are presented as mean ± SD from at least three independent experiments. Statistical comparisons between two groups were performed using a two-tail unpaired *t*-test with *p* values of < 0.05 indicating statistically significant difference. Survival data were analyzed using the Kaplan-Meier method with a log-rank (Mantel-Cox) test for comparison of survival curves. For animal studies, power analysis was performed to adjust the number of mice in each study. In general, we used sufficient animal number to obtain at least 80% power with 0.05 alpha error. For example, in the liver metastasis model, we predicted 50% of reduction in liver weight following treatment with Ly6G neutralizing antibodies. To obtain 95% power with 0.05 alpha error, we will need 3 mice in each experimental group. Synergistic effects between two drugs was calculated with Bliss independence analysis as previously reported [19]. In brief, if drug a and drug b have additive effects (independent effects), the observed effect (Y_ab-O_) should be the same as predicted (Y_ab-p_ = Y_a_ + Y_b_ − Y_a_ × Y_b_). Y_ab-p_ is the predicted effect of the combined use of drugs a and b, and Y_ab-O_ is the observed effect of the combined use of drug a and drug b. Y_a_ is suppression fraction by drug a, and Y_b_ is the suppression fraction for drug b. A more than additive effect (as synergistic effect) is predicted if Y_ab-O_ is bigger than Y_ab-p_. In contrast, antagonistic effect is predicted if Y_ab-O_ is smaller than Y_ab-p_. 

## 5. Conclusions

In conclusion, we found that combination of hedgehog (Hh) and MEK signaling inhibitors reduces pancreatic cancer metastasis in mouse models. In mouse models of pancreatic cancer metastasis, specific inhibition of smoothened signaling significantly altered the gene expression profile in the tumor microenvironment but had no significant effects on cancer metastasis. By combining Hh signaling inhibitor BMS833923 with RAS downstream MEK signaling inhibitor AZD6244, we observed reduced number of metastatic nodules in several mouse models. These two inhibitors also decreased cell proliferation significantly and reduced CD45^+^ cells (particularly Ly6G^+^CD11b^+^ cells). We demonstrated that depleting Ly6G^+^CD11b^+^ cells is sufficient to reduce cancer cell proliferation and the number of metastatic nodules. In vitro, Ly6G^+^ CD11b^+^ cells can stimulate cancer cell proliferation, and this effect is sensitive to MEK and Hh inhibition. Our results suggest that combination of smoothened inhibitor BMS833923 and MEK inhibitor AZD6244 may be effective in reducing pancreatic cancer metastasis.

## Figures and Tables

**Table 1:** Real-time PCR probe information.

Human *SHH*	HS00179843
Human *IHH*	HS01081800
Mouse *Gli1*	Mm00494654
Mouse *Ptch1*	Mm00436026
Human *GLI1*	Hs01110766

## Supplementary Materials

The following are available online at http://www.mdpi.com/2072-6694/10/11/403/s1, Figure S1: Increased expression of human GLI1 transcript in lymph node (as LN) and liver metastases (as Liver), Figure S2: Effects of K-RAS downstream signaling inhibitors on GLI1 expression in cancer cells, Figure S3. Detection of microscopic and macroscopic metastases by intravital microscope, Figure S4: Whole body fluorescent images of GFP-expressing AsPC1 cancer cells in mice with different treatments, Figure S5: Primary tumor weight in different treatment groups, Figure S6: Effects of AZD6244 and BMs833923 on target protein expression, Figure S7: Gene expression analysis of metastatic niche, Figure S8: Cell population analyses of metastatic liver tissues, Figure S9: Cell population changes by MEK/Hh signaling inhibition, Figure S10: Effects of 1A8 on Ly6G cell population.
