# Supplementary material for: Simultaneous Inhibition of MEK and Hh Signaling Reduces Pancreatic Cancer Metastasis
Source: Cancers (Basel). 2018 Oct 26;10(11):403. doi: 10.3390/cancers10110403 (PMC6266431; doi:10.3390/cancers10110403)
Supplement: Supplementary file 1 [file cancers-10-00403-s001.pdf]

Supplementary Materials: Simultaneous Inhibition of MEK and Hh Signaling Reduces Pancreatic Cancer Metastasis

Dongsheng Gu, Hai Lin, Xiaoli Zhang, Qipeng Fan, Shaoxiong Chen, Safi Shahda, Yunlong Liu, Jie Sun and Jingwu Xie

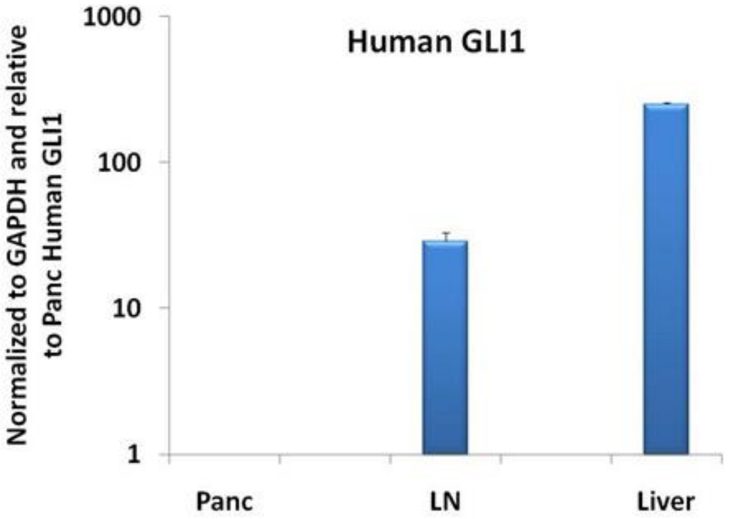

Figure S1. Increased expression of human GLI1 transcript in lymph node (as LN) and liver metastases (as Liver). MIA-PaCa2 cells were injected into pancreas of immune deficient NSG mice. When tumor metastases are evident, we harvested pancreatic (as Panc), lymph node and livertissues to extract total RNAs for gene expression analyses. We used GAPDH as the internal control, and human GLI1 as a readout for hedgehog signaling. ** indicates $p < 0.05$.

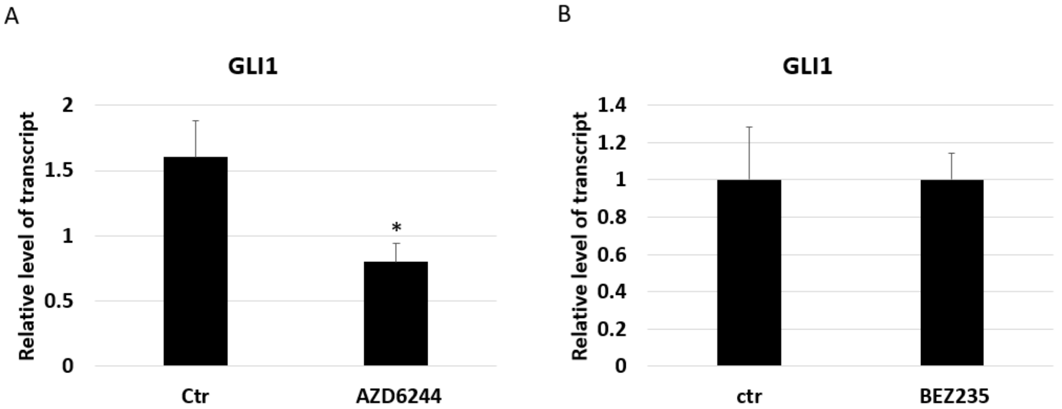

Figure S2. Effects of K-RAS downstream signaling inhibitors on GLI1 expression in cancer cells. We treated human cancer cells (AsPC1) with MEK inhibitor AZD6244 and PI3K/mTOR dual inhibitor BEZ235 overnight. Harvested cells were used to extract total RNAs and to analyze gene expression. We used GAPDH as the internal control to measure the level of GLI1 transcript, a marker for hedgehog signaling activation. We have triplicated samples for each group, and the experiment was repeated 2 times. * indicate statistically significant difference ($p < 0.05$).

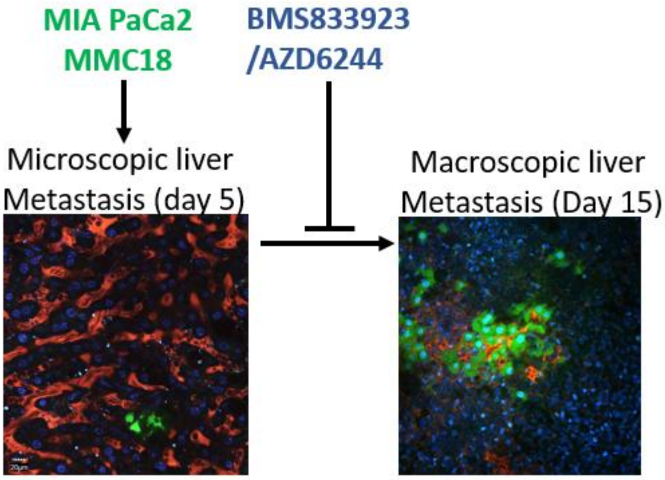

Figure S3. Detection of microscopic and macroscopic metastases by intravital microscope. Cancer cells labeled with GFP were injected into pancreas. Dynamic movement of liver cells were visualized by intravital microscope. Vasculature was visualized by rhodamine-labeled dextran. Cell nucleus was visualized by Hoescht 33342. GFP indicates cancer cells. Scale: 200 ×.

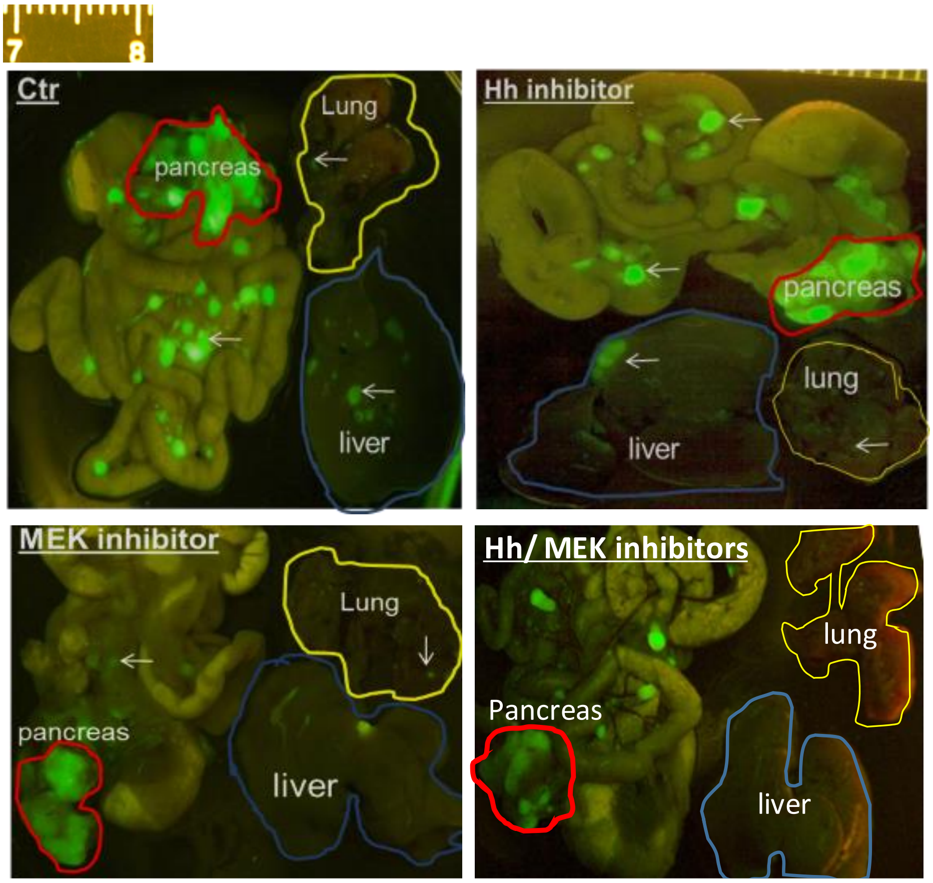

Figure S4. Whole body fluorescent images of GFP-expressing AsPC1 cancer cells in mice with different treatments. Mice were sacrificed to obtain liver, lung pancreas and intestine for whole body imagining of GFP- expressing cancer cells. In the control group, cancer cells were detected in all these tissues. In both BMS833923 and AZD6244- treated groups, pancreatic tumors were significantly reduced. In combined treatment, no GFP-expressing cells were detected in lung and liver tissues. By histology, we only detected cancer cells in 3 out of 8 lung tissues, indicating significant reduction in lung metastasis. Scale bar: cm

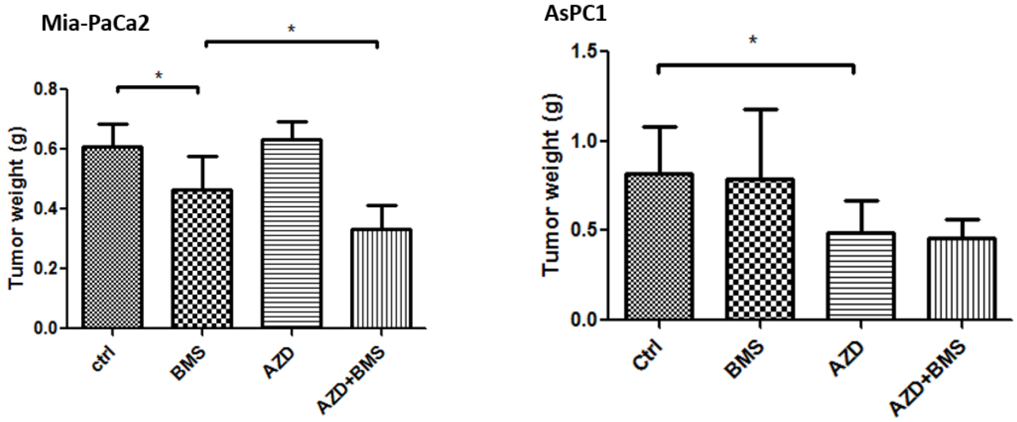

Figure S5. Primary tumor weight in different treatment groups * indicates statistical significance in comparison between two groups ($p < 0.05$). Combined treatment with MEK inhibitor AZD6244 (as AZD) and smoothened signaling inhibitor BMS833923 (as BMS), however, did not achieve a more than additive effect (via BLISS independent analysis).

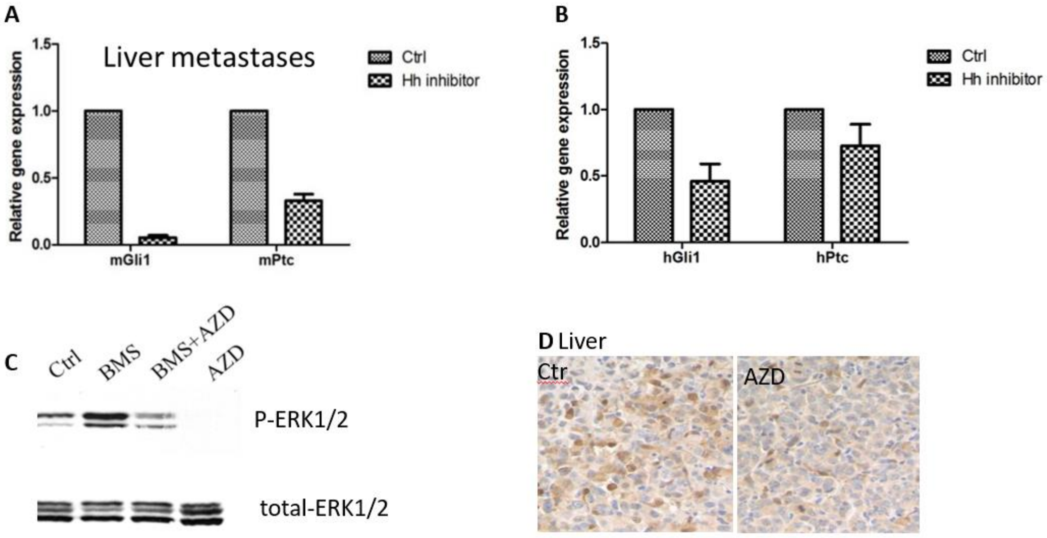

Figure S6. Effects of AZD6244 and BMs833923 on target protein expression. (**A**) shows reduced expression of hedgehog target genes Gli1 and Ptch1 in the liver tissue after BMS833923 (as Hh inhibitor) treatment. (**B**) shows incomplete suppression of GLI1 (as hGli1) and PTCH1 (as hPtc) in metastatic liver tissues. (**C**) Detection of p-ERK1/2 by Western blotting in liver tissues treatment with different drugs. AZD6244 (as ASZ) was effective in reducing the level of phosphor-ERK. (**D**) detection of p-ERK1/2 by immunohistochemistry in liver tissues treated with AZD6244. Please note that the elevated level of p-ERK1/2 observed in BMS-treated mouse (shown in **C**) was not observed in other mice. Scale: 400 ×.

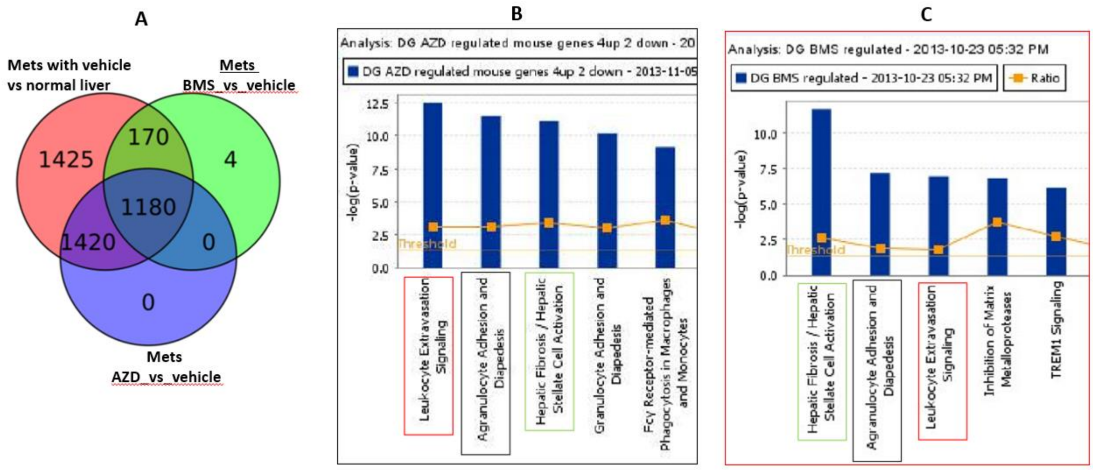

Figure S7. Gene expression analysis of metastatic niche. Gene expression from liver metastatic tissues with different treatments (BMS833923, shown as BMS; AZD6244 shown as AZD) was profiled using RNA-sequencing. (**A**) shows shared genes affected by both BMS833923 and AZD6244. (**B**) shows pathways affected both AZD6244 and BMS833923. Please note that the top 3 pathways were shared by both BMS833923 treatment and AZD6244 treatment.

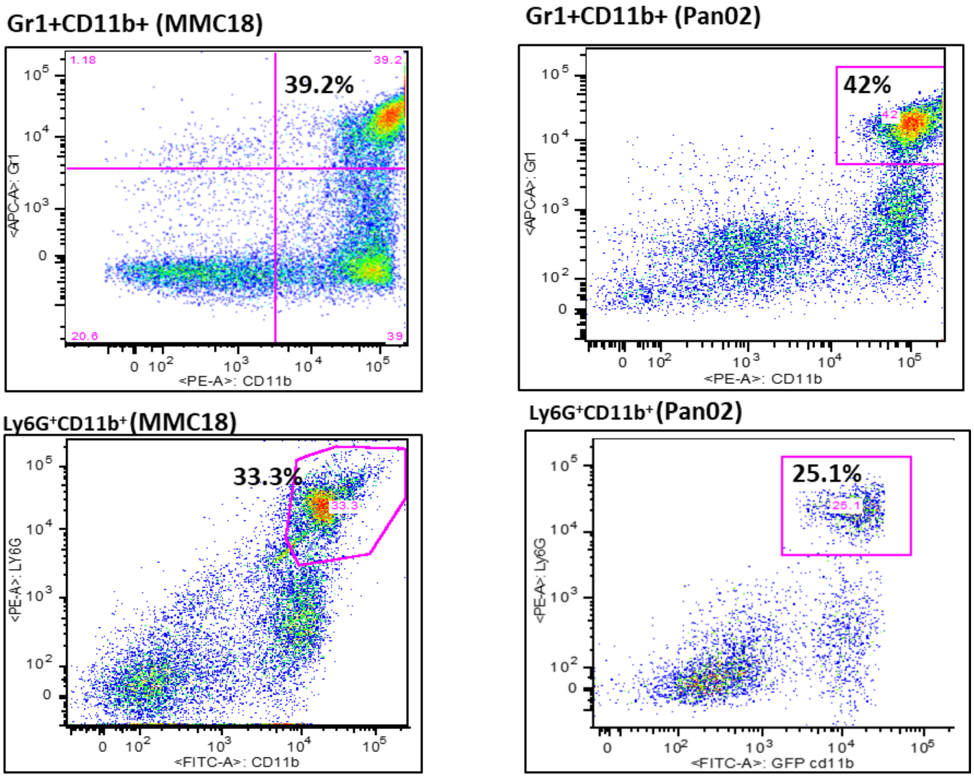

Figure S8. Cell population analyses of metastatic liver tissues. $CD11b^+Gr1^+$ cells were a major cell population in metastatic niche, and $Ly6G^+$ cells is the major cell type, contributing to >60% of the $CD11b^+Gr1^+$ cells.

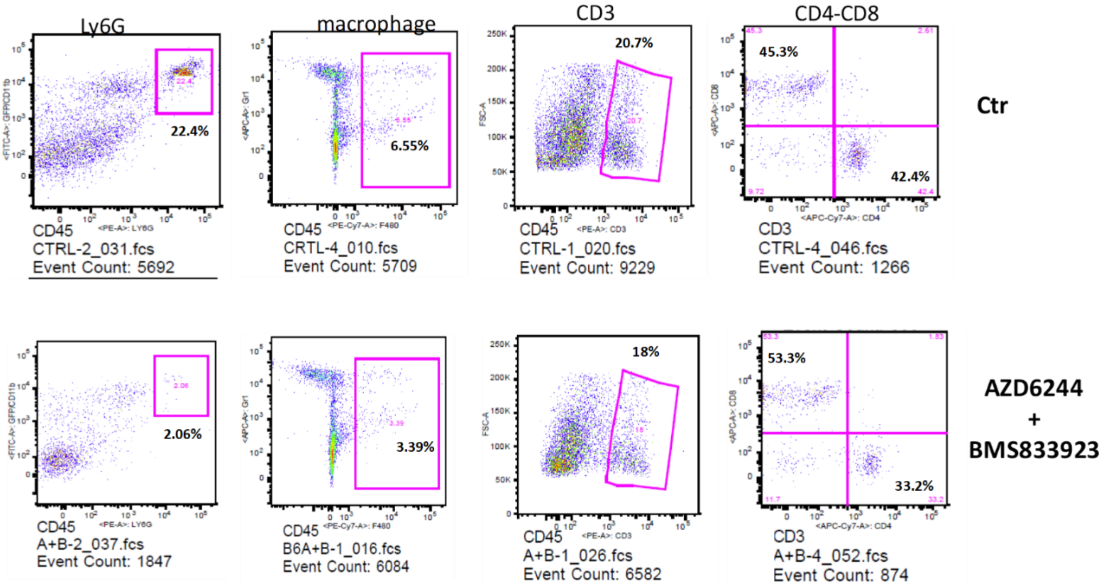

Figure S9. Cell population changes by MEK/Hh signaling inhibition. C57B/6 mice with MMC18-derived tumors treated with AZD6244 and BMS833923 were analyzed for cell. population changes in the metastatic niche (CD45⁺ cells). We noticed that the combined treatment reduced Ly6G⁺CD11b⁺ cells by 90%, and increased CD8 T cells by 9%.

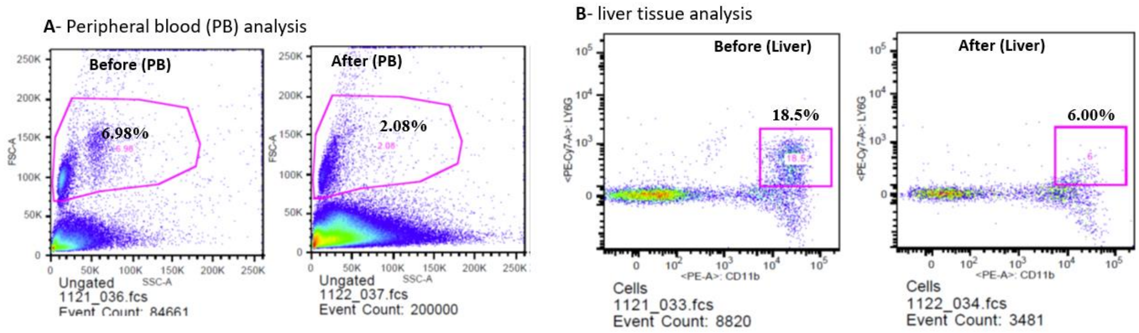

Figure S10. Effects of 1A8 on Ly6G cell population**.** We analyzed peripheral blood and liver tissues before and after. administration of Ly6G neutralizing antibodies. (**A**) Using FSC and SSC, we noticed a population (highlighted) was reduced from 6.98% to 2.08%, which was shown to be Gr1⁺CD11b⁺ cells (shown in B). (**B**) shows reduced CD11b⁺Ly6G⁺ cells in the liver tissues after injection of 1A8 IgG which depletes Ly6G⁺ CD11b⁺ cells. We found a reduction of this population from 18.5% to 6% after depletion. PB: peripheral blood.
